# Coinfection of Hepatitis B and Hepatitis C Virus in Patients With Human Immunodeficiency Virus

**DOI:** 10.7759/cureus.16474

**Published:** 2021-07-19

**Authors:** Hassan Masroor, Usman M Qazi, Anum Masroor, Ayesha Saleem, Ghayyur Khalil, Kiran Abbas

**Affiliations:** 1 Psychiatry and Behavioral Sciences, Psychiatric Care Associates, Peshawar, PAK; 2 Internal Medicine, Khushal Medical Center, Peshawar, PAK; 3 Medicine, Khyber Medical College, Peshawar, PAK; 4 General Surgery, Hayatabad Medical Complex, Peshawar, PAK; 5 Respiratory Medicine, Glenfield Hospital, Glenfield, GBR; 6 Medicine, Jinnah Postgraduate Medical Centre, Karachi, PAK

**Keywords:** hbv, hiv aids, human immunodeficiency virus infection, hepatitis, coinfection

## Abstract

Background

Coinfection of viral hepatitis and human immunodeficiency virus (HIV) is not uncommon in Pakistan. Coinfection of hepatitis B virus (HBV) and hepatitis C virus (HCV) with HIV is associated with a poor prognosis. The current study evaluated the occurrence of coinfection of HBV/HIV and HCV/HIV in Peshawar, Khyber Pakhtunkhwa, Pakistan.

Methodology

A prospective, observational study was conducted at Khushal Medical Center and Hayatabad Medical Complex (HMC) between February 2019 and April 2020. All patients with confirmed HIV positive serum samples aged above 18 years were eligible to apply for anonymous screening for hepatitis B and C virus markers. Sociodemographic data including patient’s age, gender, marital status, occupation, employment status, and body mass index among others were documented on a predefined proforma. The presence of viral markers of HBV and HCV in HIV patients was the primary outcome of the study.

Results

Out of the total of 650 HIV patients, 78 (12%) had coinfection with hepatitis virus. The mean age was 42.40 ± 10.96 years. Sixty-three (80.77%) patients had coinfection with hepatitis B virus infection while 15 (19.23%) had hepatitis C coinfection. No cases of triple infections were identified. It was found that patients infected with HIV/HBV were more frequent in the age group of 30 to 45 years (36; 85.71%) while the HIV/HCV patients were older, i.e. 72.72% were older than 45 years (p<0.001). The sexual route was strongly associated with HIV/HBV group compared to HIV/HCV group [51 (89.47%) vs. six (10.53%); p<0.0001].

Conclusion

The current study highlighted the rate of coinfection of HBV and HCV in HIV-infected individuals in Pakistan. We found that four-fifths of patients had coinfection with HBV while only one-fifth had coinfection with HCV. These findings are consistent with the published literature revealing that HIV/HBV and HIV/HCV are common in developing countries. Young sexually active individuals are at a significantly higher risk of acquiring HIV/HBV infection than HIV/HCV. We advocate screening for these hepatitis viral markers in patients with HIV infection as well as their sexual partners. Further large-scale, multicentre, and multistate studies should be conducted to determine the burden of these communicable diseases.

## Introduction

Worldwide, two of the most common chronic viral infections documented are the hepatitis B virus (HBV) and hepatitis C virus (HCV) [1]. Since hepatitis and human immunodeficiency virus (HIV) have the same routes of transmission, i.e. sharing of intravenous needles, blood, and unsafe sexual activity, it is not uncommon to observe a coinfection of hepatitis in patients with HIV [2]. 

Coinfections of HBV and HCV in HIV-positive patients can greatly affect an individual's quality of life and are associated with a poor prognosis [3]. HBV and HIV are epidemiologically similar [4]. Anti-HBS (marker which signifies past infection of HBV) or HBsAg (a marker of the surface antigen of hepatitis B) are shown to be positive in HIV patients since 10% of patients with HIV have been documented to have a history of hepatitis B [4,5].

A study by Mohammadi et al. revealed that in 94.4% of HIV patients, hepatitis viruses were detected [6]. In 14.5%, viral markers for HBsAg were positive while in 72% anti-HCV was positive. The authors also revealed triple infections in 7.9% of cases. Hepatitis B infections are very common and a global health problem especially in the subcontinent [7,8]. ﻿Gupta et al. revealed the prevalence of HBsAg to be 5.3% in patients with HIV and the rate of HCV coinfection with HIV of 2.43% [9]. The study further confirmed that triple infection cases were not detected in the population. Due to the scarce data present on the epidemiology of coinfection of HBV and HCV in patients with HIV in our local population, the current study was undertaken to fill the literature gap.

## Materials and methods

A prospective, observational study was conducted in the outpatient departments at Khushal Medical Center and Hayatabad Medical Complex (HMC) between February 2019 to April 2020. Prior to the initiation of the study, ethical approval was obtained from the concerned department. A non-probability convenience sampling technique was used to recruit participants in the study. Hayatabad Medical Complex (HMC) issued approval IRB#137-412-2020.

All patients with confirmed HIV positive serum samples aged above 18 years were eligible to apply for anonymous screening for hepatitis B and C virus markers. Those patients who were below 18 and refused participation were excluded from the study. Informed consent was obtained from all patients prior to the study. For screening of hepatitis B virus and C virus, enzyme-linked immunosorbent assay (ELISA) kits were utilized. For HBV, hepatitis B surface antigen (HBsAg) was detected and for HCV, antibodies to hepatitis C virus (anti-HCV) were determined. To establish the diagnosis, polymerase chain reaction (PCR) for HBV-DNA and reverse transcriptase-nested polymerase chain reaction (RT-PCR) for HCV-RNA were done [10,11].

Sociodemographic data including patient’s age, gender, marital status, occupation, employment status, and body mass index among others were documented on a predefined proforma. The presence of viral markers of HBV and HCV in HIV patients was the primary outcome of the study.

All the data were analyzed using SPSS version 26 (IBM Corp., Armonk, NY, USA). For continuous variables, mean and standard deviation were determined. For categorical variables, the frequency was determined. All the data was presented in tabular and graphical form. The Chi-Square test was applied to determine the significant variation between groups with respect to different sociodemographic and clinical variables. A p-value of below 0.05 was the cut-off for statistical significance. 

## Results

Out of the total of 650 HIV patients, 78 (12%) had coinfection with hepatitis virus. There was a dominance of the male gender as compared to females (91.03% vs 7.69%). A mean age of 42.40 ± 10.96 years was documented. The majority were between 30 and 45 years. Sixty-three (80.77%) patients had coinfection with hepatitis B virus while 15 (19.23%) had hepatitis C coinfection. No cases of triple infections were identified. The demographics of the patients are presented in Table 1.

**Table 1:** Sociodemographic Variables of Study Participants and Virological Outcomes

Characteristics	n (%)
Age Groups	
<30	25 (32.05%)
30-45	42 (53.85%)
>45	11 (14.10%)
Gender	
Male	71 (91.03%)
Female	7 (8.97%)
Marital Status	
Married	56 (71.79%)
Unmarried	14 (17.95%)
Widowed	4 (5.13%)
Separated/Divorced	4 (5.13%)
Coinfection with Hepatitis Markers	
Detected	78 (12.00%)
Not Detected	572 (88.00%)
Co-infected with HBV	
Yes	63 (80.77%)
No	15 (19.23%)
Co-infected with HCV	
Yes	15 (19.23%)
No	63 (80.77%)

Age was significantly associated with the coinfection of viral markers in HIV patients. It was found that patients infected with HIV/HBV were more frequent in the age group 30-45 years (36; 85.71%) while the HIV/HCV patients were older, i.e. 72.72% were older than 45 years (p<0.001). The suspected route of transmission in 73.18% of cases was sexual. This was followed by intravenous drugs (11; 14.1%). The sexual route was strongly associated with HIV/HBV group compared to HIV/HCV group [51 (89.47%) vs. six (10.53%); p<0.0001]. HIV/HCV was more common (42.85%) in females while HIV/HBV was more frequent in males (59; 83.11%). However, the difference did not vary significantly between the two infections (p=0.096) (Table 2). 

**Table 2:** Distribution of Age, Gender, and Routes of Transmission With Respect to the Viral Hepatitis

	Total	Groups		P-value
Characteristics	n (%)	HIV + HBV	HIV + HCV	
Age Groups				
<30	25 (32.05%)	24 (96.00%)	1 (4.00%)	<0.0001
30-45	42 (53.85%)	36 (85.71%)	6 (14.29%)	
>45	11 (14.10%)	3 (27.27%)	8 (72.72%)	
Gender				
Male	71 (91.03%)	59 (83.11%)	12 (11.26%)	0.096
Female	7 (8.97%)	4 (57.14%)	3 (42.85%)	
Suspected Route of Transmission				
Sexual	57 (73.18%)	51 (89.47%)	6 (10.53%)	<0.0001
Intravenous Drugs	11 (14.10%)	3 (27.27%)	8 (72.73%)	
Hematological	7 (8.97%)	3 (42.86%)	4 (57.14%)	
Unknown	3 (3.84%)	2 (66.67%)	1 (33.33%)	

## Discussion

The current study aimed to highlight the rate of coinfection of HBV and HCV in HIV-infected individuals in Khyber Pakhtunkhwa, Pakistan. We found that out of the 78 cases of HIV and hepatitis coinfection, four-fifths had HBV and the remainder had HCV coinfection.

Our study findings were in accordance with the published studies [1,12-17]. Sharma et al. revealed that HBV/HIV infection was found in 11% while HCV/HIV was found in 13% of patients. However, the data was collected in 2011 and was a single-center study, thus limiting interference from the study [12]. According to the statistics reported in the year 2020 by the Department of Epidemiology of Iran, the trend of HBV and HCV is on the rise with the current incidence rate of infections at 13.66% and 5.46% per 100,000, respectively [13]. Similar to the current study, the Iranian study authored by Soori et al. also reported a predominance of males. Chen et al. reported the rates of HBV/HIV and HCV/HIV coinfection as 7.2% and 50.2% whereas in 12.2% of individuals triple infections were reported [14]. 

HIV is a dreadful disease and results in Acquired Immunodeficiency Syndrome (AIDS); however, coinfection with either HBV or HCV further deteriorates the chances of survival [1]. It may lead to complications such as cirrhosis or hepatocellular carcinoma. A study from Guangxi, China, revealed that 48.67% of patients with seropositive HIV also had HCV. Furthermore, the highest rate of anti‑HCV positivity was found in drug users [15]. Since HCV and HIV share similar transmission routes (blood transfusions, unprotected sexual intercourse, intravenous drug users, parenteral routes, perinatal routes (mother to child), and hemodialysis) coinfection of HIV/HCV is more common than HBV/HCV [16]. However, in our study, HBV/HIV was more frequently observed which could be related to the more infectious nature of HBV than HCV [17]. 

It is advocated to screen for viral hepatitis in patients with HIV irrespective of their age. Our study found that HBV/HIV was more common in patients aged 35 to 45 years while HCV/HIV was more common in patients older than 45 years. We also found that HCV/HIV was predominant in the male population aged 20 to 40 years [15].

Pakistan is a developing country and hence has limited resources to rehabilitate drug addicts with HIV and improve their quality of life. With a population of over 225 million, we have a limited capacity with respect to both the health and education sectors. Patients with HIV need proper counseling and awareness about their disease so that the patients understand the catastrophic result of their continuous use of IV drugs and engagement in unsafe sexual intercourse. We need to develop strategies regarding implementing effective health promotion programs in shanty settlements across the country.

The current study significantly contributes to the existing literature; however, there are some limitations. It was a hospital-based study, therefore, does not truly represent the entire population. In order to truly understand the burden of HIV and coinfection with hepatitis B and C, we must conduct large-scale, multicentre, and multistate studies across Pakistan.

## Conclusions

The current study highlighted the rate of coinfection of HBV and HCV in HIV-infected individuals in Pakistan. We found that four-fifths of patients had coinfection with HBV while only one-fifth had coinfection with HCV. These findings are consistent with published literature revealing that HBV/HIV and HCV/HIV are common in developing countries. Young sexually active individuals were significantly at a higher risk of acquiring HBV/HIV coinfection than HCV/HIV. We advocate screening for these hepatitis viral markers in patients with HIV infection as well as their sexual partners. Further large-scale, multicentre, and multistate studies should be conducted to determine the burden of these communicable diseases. 
